# Supplementary material for: The interactome of intact mitochondria by cross-linking mass spectrometry provides evidence for coexisting respiratory supercomplexes
Source: Mol Cell Proteomics. 2017 Dec 8;17(2):216–32. doi: 10.1074/mcp.RA117.000470 (PMC5795388; doi:10.1074/mcp.RA117.000470)
Supplement: Supplemental Data [file supp_RA117.000470_133922_0_supp_23978_fzffwf.zip › spectra_annotation/mito_spectra_annotation/415-1-3-1-3-1.pdf]

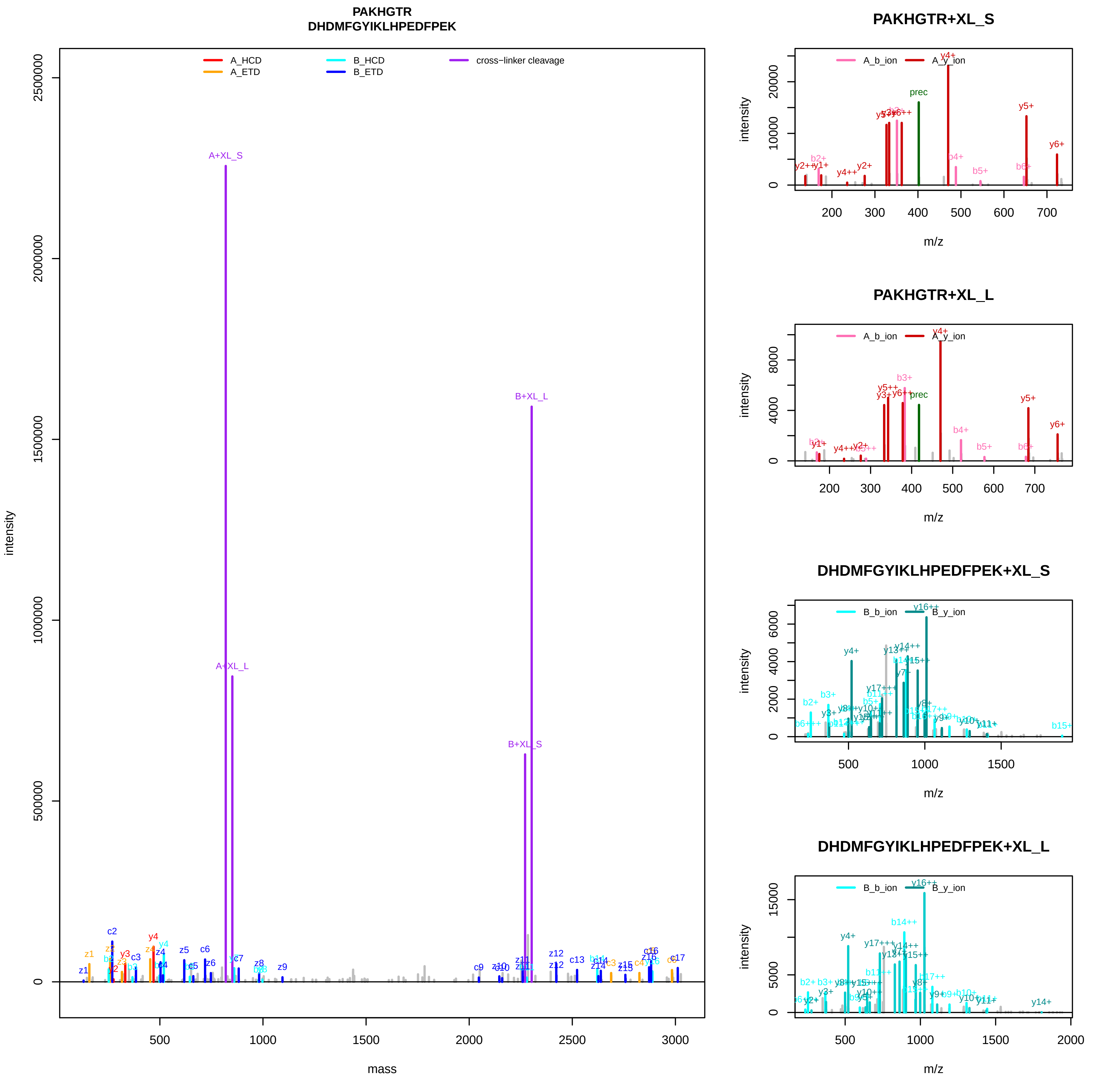
PAKHGTR
DHDMFGYIKLHPEDFPEK
A_HCD
A_ETD
B_HCD
B_ETD
cross−linker cleavage
A+XL_S
A+XL_L
B+XL_L
B+XL_S
intensity
mass
0
500000
1000000
1500000
2000000
2500000
500
1000
1500
2000
2500
3000
PAKHGTR+XL_S
A_b_ion
A_y_ion
prec
y4+
y5+
y6+
m/z
PAKHGTR+XL_L
A_b_ion
A_y_ion
prec
b3+
b4+
b5+
m/z
DHDMFGYIKLHPEDFPEK+XL_S
B_b_ion
B_y_ion
y16++
y4+
b15+
m/z
DHDMFGYIKLHPEDFPEK+XL_L
B_b_ion
B_y_ion
y16++
b14++
y4+
y14+
m/z
